# Supplementary material for: Linker histones consolidate heterogenous nucleosome fiber contacts by linking together multiple nucleosomes
Source: Nat Commun. 2026 Mar 5;17:3807. doi: 10.1038/s41467-026-69842-x (PMC13111611; doi:10.1038/s41467-026-69842-x)
Supplement: Supplementary file 1 — Supplemetary Information [file 41467_2026_69842_MOESM1_ESM.pdf]

Linker Histones Consolidate Heterogenous Nucleosome Fiber Contacts by Linking Together Multiple Nucleosomes

SUPPLEMENTARY INFORMATION

Zenita Adhireksan, Deepti Sharma, Qiuye Bao, Phoi Leng Lee, Sivaraman Padavattan,

Gabriela E. Davey, Jeffrey C. Hansen & Curtis A. Davey

CONTENTS

4 Tables: pages 2–5

17 Figures: pages 6–27

Supplementary Table 1. Data collection and refinement statistics for H1x, H5, and H1.3 assemblies

	**H1x^{L1}-169a**[a]	**H5$^{D/L2}$-169a**	**H1.3^{F}-169a**
**Data collection**			
Space group	$P2_1$	$P2_1$	$P2_1$
Cell dimensions			
a, *b*, *c* (Å)	104.85, 102.11, 216.18	105.16, 103.76, 212.96	105.07, 101.74, 218.40
α, β, γ (°)	90, 96.34, 90	90, 100.97, 90	90, 103.52, 90
Resolution (Å)	2.70-92.2 (2.70–2.85)[b]	2.84-48.0 (2.84–2.99)[b]	3.19-47.2 (3.19–3.37)[b]
R_{merge} (%)	5.9 (93.0)	7.1 (165.9)	7.3 (71.7)
R_{pim} (%)	4.4 (68.1)	3.2 (78.4)	5.5 (56.2)
$I / \sigma I$	9.8 (1.1)	13.7 (1.0)	10.2 (1.4)
CC½ (%)	99.8 (52.1)	99.9 (42.1)	99.8 (53.2)
Completeness (%)	99.6 (97.8)	98.8 (92.4)	98.5 (96.1)
Redundancy	3.4 (3.5)	6.6 (6.1)	3.3 (3.2)
**Refinement**			
Resolution (Å)	2.70–92.2	2.84–48.0	3.19–47.2
No. reflections	121,849	103,041	69,849
$R_{\mathrm{work}}/R_{\mathrm{free}}$ (%)	21.0 / 26.7	20.0 / 25.9	20.3 / 25.9
No. atoms	28,083	27,693	26,617
Core histone	12,557	12,516	12,198
Linker histone	1,455	1,297	560
DNA	13,846	13,846	13,846
Solvent	225	34	13
B-factors (Å^2)	95	121	128
Core histone	70	97	103
Linker histone	134	164	180
globular domain	131	159	181
tail residues	145	197	159
DNA	114	140	149
Solvent	72	102	114
R.m.s. deviations			
Bond lengths (Å)	0.005	0.008	0.007
Bond angles (°)	1.23	1.30	1.25

[a] Note that H1x^{L1}-338b data/statistics were presented earlier in Adhireksan *et al.*, 2021 (ref. 21)

[b] Single crystal data sets; Data collection values in parentheses are for the highest-resolution shell

Supplementary Table 2. Data collection and refinement statistics for H1.0 assemblies

	$H1.0^{L1}$-169a[a]	$H1.0^{L1b}$-169a	$H1.0^{L2b}$-169an
**Data collection**			
Space group	$P2_1$	$P2_1$	$P2_1$
Cell dimensions			
a, *b*, *c* (Å)	104.80, 102.76, 218.05	208.78, 102.37, 212.71	213.85, 102.46, 218.29
α, β, γ (°)	90, 97.40, 90	90, 101.12, 90	90, 100.5, 90
Resolution (Å)	3.20-39.9 (3.20–3.37)[b]	3.39-48.8 (3.39–3.57)[b]	3.51-88.3 (3.51–3.57)[b]
R_{merge} (%)	10.8 (81.1)	8.6 (150.7)	12.4 (182.1)
R_{pim} (%)	8.4 (61.2)	3.9 (68.0)	5.4 (81.1)
$I / \sigma I$	5.3 (1.2)	10.5 (1.1)	8.0 (1.1)
CC½ (%)	99.3 (55.7)	100.0 (59.8)	100.0 (37.0)
Completeness (%)	99.3 (96.6)	98.9 (93.4)	100.0 (100.00)
Redundancy	3.0 (2.8)	6.7 (6.7)	6.1 (5.9)
**Refinement**			
Resolution (Å)	3.20–39.9	3.39–48.8	3.51–88.3
No. reflections	74,093	119,573	110,177
$R_{\mathrm{work}}/R_{\mathrm{free}}$ (%)	20.1 / 26.2	22.7 / 29.6	20.6 / 27.9
No. atoms	27,249	53,518	52,574
Core histone	12,136	24,624	24,255
Linker histone	1,243	1,149	595
DNA	13,846	27,692	27,724
Solvent	24	53	--
B-factors ($Å^2$)	126	178	160
Core histone	93	148	123
Linker histone	179	237	199
globular domain	179	237	198
tail residues	175	229	213
DNA	149	201	191
Solvent	110	151	--
R.m.s. deviations			
Bond lengths (Å)	0.006	0.008	0.006
Bond angles (°)	1.29	1.30	1.35

[a] Note that this $H1.0^{L1}$-169a data set was also presented earlier in Adhireksan *et al.*, 2021 (ref. 21)

[b] Single crystal data sets; Data collection values in parentheses are for the highest-resolution shell

Supplementary Table 3. Kinetic/affinity measurements of linker histone association with the 169a-nucleosome, based on bio-layer interferometry

	k_a ($s^{-1}M^{-1}$)[a]	k_d (s^{-1})	K_D (M)
H1.3	$2.93\pm0.01 \times 10^{6}$	$4.25\pm0.08 \times 10^{-5}$	$1.45\pm0.03 \times 10^{-11}$
H1.0	$3.36\pm0.01 \times 10^{6}$	$6.28\pm0.07 \times 10^{-5}$	$1.87\pm0.02 \times 10^{-11}$
H5[b]	$1.55\pm0.00 \times 10^{6}$	$5.25\pm0.03 \times 10^{-5}$	$3.39\pm0.02 \times 10^{-11}$
H1x	$3.68\pm0.02 \times 10^{6}$	$1.64\pm0.01 \times 10^{-4}$	$4.46\pm0.03 \times 10^{-11}$

[a] See Supplementary Fig. 7 for the corresponding sensorgrams and fitted results.
[b] Note the binding affinity for H5 may be underestimated due to the absence of 43 residues from the C-terminus.

Supplementary Table 4. Interfacial areas associated with the linker histone globular domain-DNA contacts

Assembly	LH mode	ΔASA[a] (total)	ΔASA (polar)	ΔASA (apolar)
169a-H5	D	2,199	1,466	733
169a-H5	L2	2,188	1,338	850
349c-H1.0	D	1,569	1,161	407
349c-H1.0	D (1N)[b]	1,425	1,043	382
349c-H1.0	L2	1,534	1,088	446
169an-H1.0	L2b	1,366	934	432
169a-H1.0	L1	1,366	873	463
169a-H1.0	L1 (1N)[b]	783	517	266
169a-H1.0	L1b	849	669	180
169a-H1x	L1	1,209	854	355
169a-H1x	L1 (1N)[b]	819	579	240
169a-H1.3	F	1,562	1,027	535
169a-H1.3	F (1N)[b]	982	573	409

[a] ΔASA, change in solvent accessible surface area ($Å^2$)
[b] ΔASA calculations based on the contact area associated with only a single nucleosome (1N). This single nucleosome is either the host nucleosome (D/F modes) or that involving the major interface (L1 mode).

a

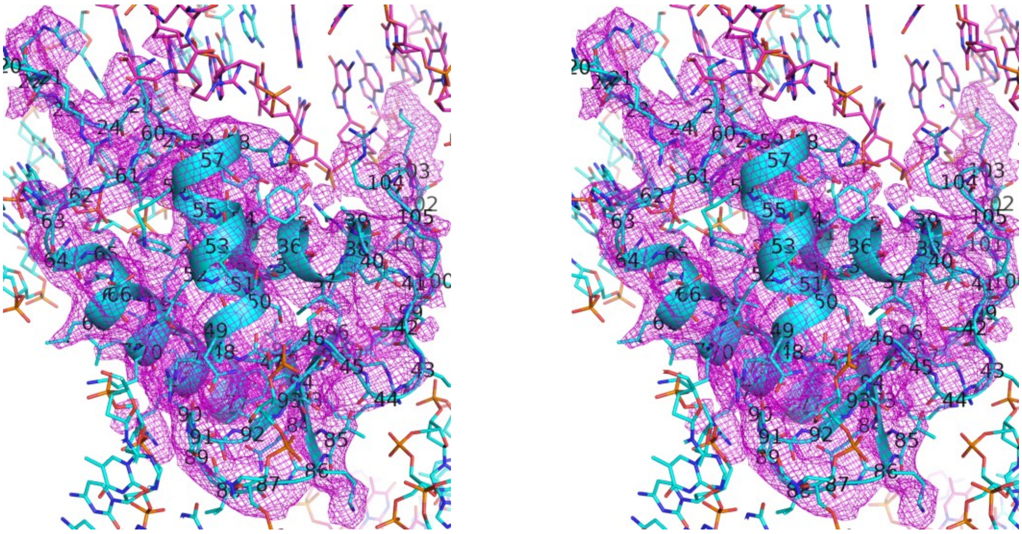

b

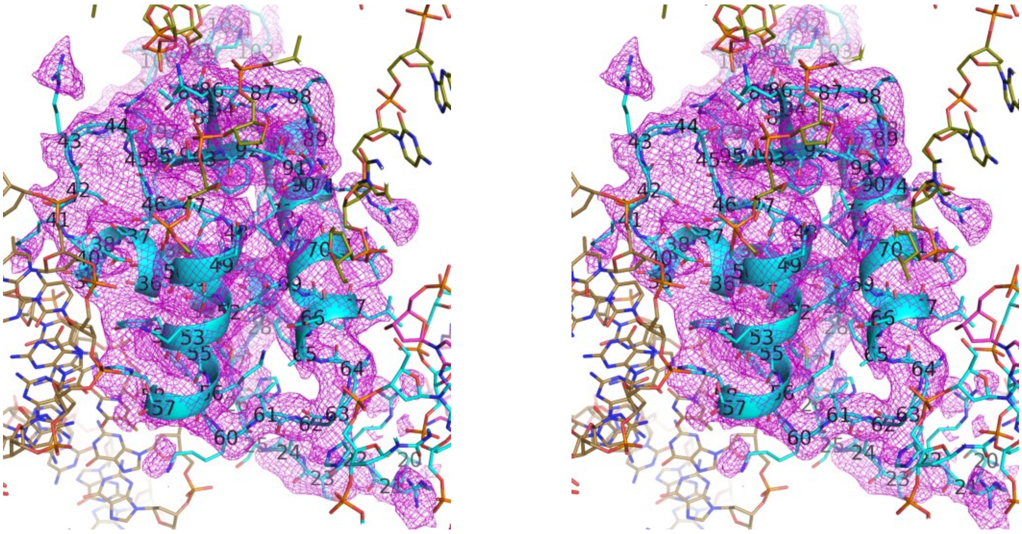

Supplementary Figure 1. Experimental electron density corresponding to the LHs in the H5-169a structure, in stereo view. An F_O-F_C omit electron density map (magenta; contoured at 1σ; LH atoms omitted from the model) in the vicinity of the omitted atoms is superimposed onto the refined model. (**a**,**b**) The two views correspond to LH1 (a; on-dyad [D] mode) and LH2 (b; linking [L2] mode).

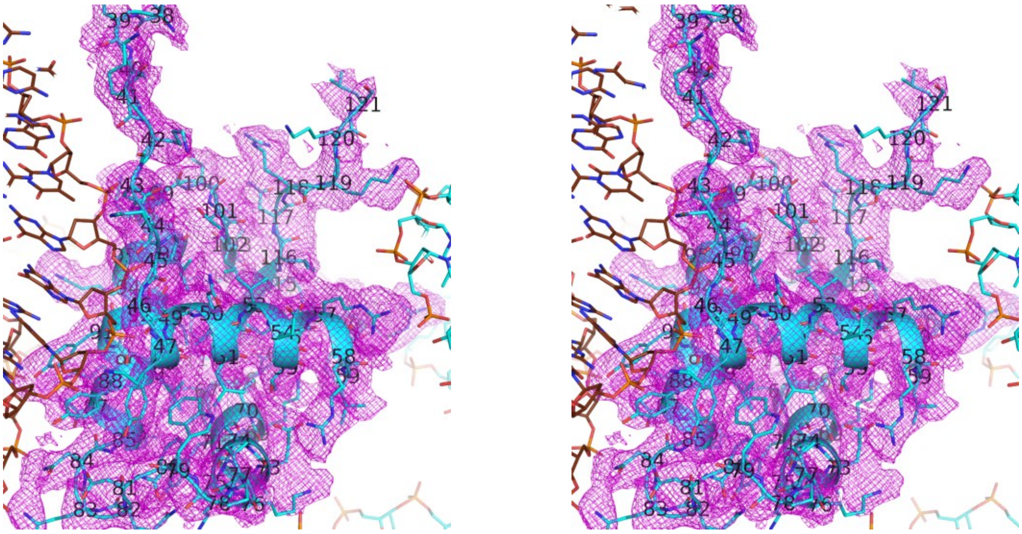

Supplementary Figure 2. Experimental electron density corresponding to the LH (linking [L1] mode) in the H1x-338b structure, in stereo view. An $F_O - F_C$ omit electron density map (magenta; contoured at 1.0σ; LH atoms omitted from the model) in the vicinity of the omitted atoms is superimposed onto the refined model. This H1x-338b structure is from Adhireksan *et al.*, 2021 (*pdb* code 6L9Z; ref. 21).

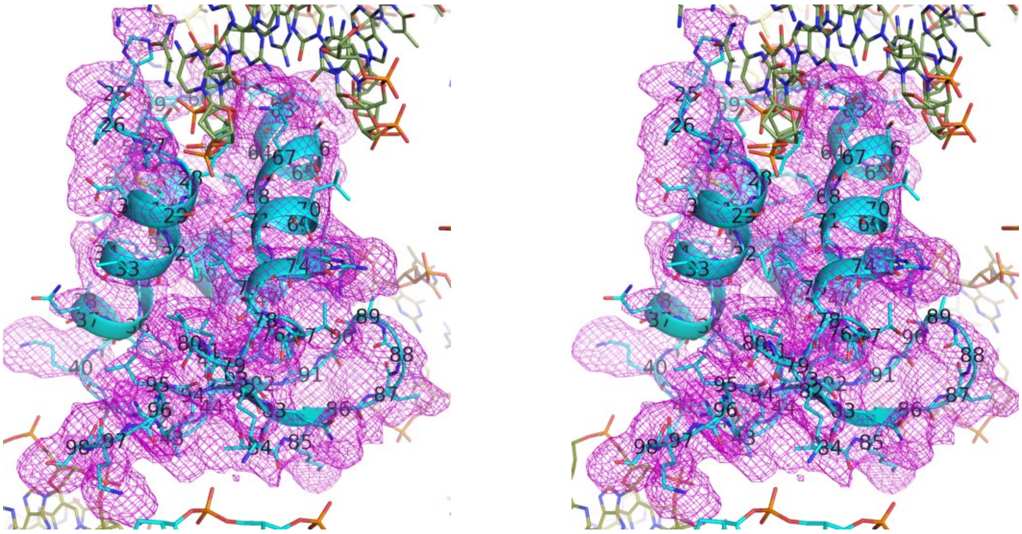

Supplementary Figure 3. Experimental electron density corresponding to LH1 (linking [L1b] mode) in the H1.0-169a structure, in stereo view. An F_O-F_C omit electron density map (magenta; contoured at 0.5σ; LH1 atoms omitted from the model) in the vicinity of the omitted atoms is superimposed onto the refined model.

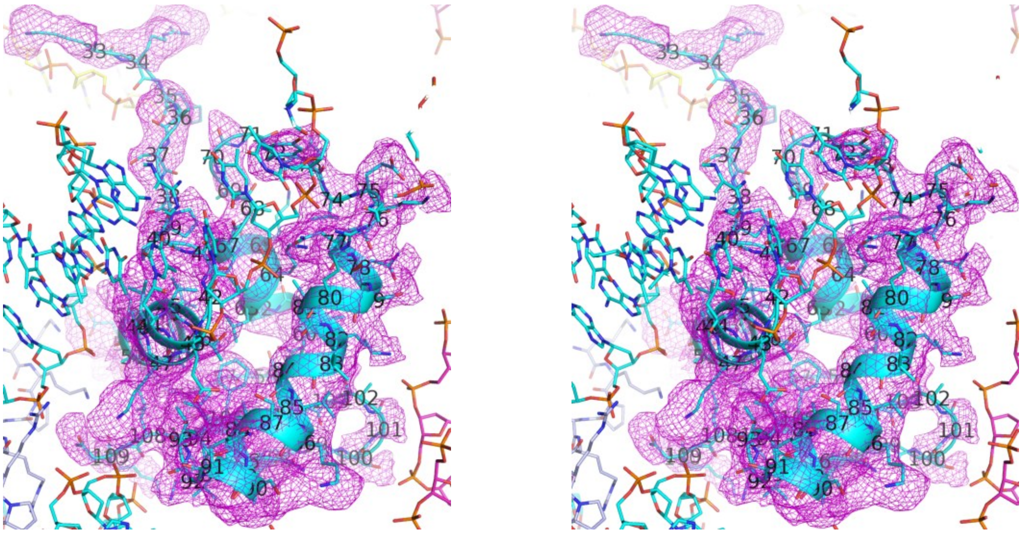

Supplementary Figure 4. Experimental electron density corresponding to the LH (off-dyad [F] mode) in the H1.3-169a structure, in stereo view. An F_O-F_C omit electron density map (magenta; contoured at 0.5σ; LH atoms omitted from the model) in the vicinity of the omitted atoms is superimposed onto the refined model.

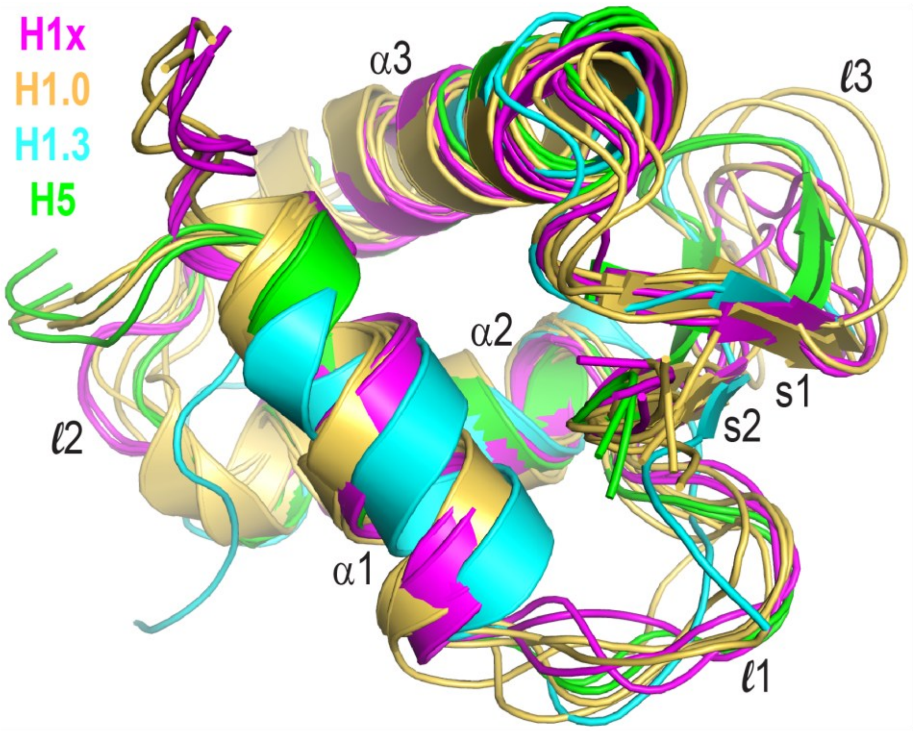

Supplementary Figure 5. Conformational variability in the linker histone globular domain. Superimposition of the LH_{GD} regions from the 169a (*pdb* codes 8YTI $[H1x]_2$, 6LAB $[H1.0]_2$ [ref. 22], 7XX6 $[H1.0]_2$, 7XX5 [H1.3], 7XVM $[H5]_2$), 169an (*pdb* code 7XVL [H1.0]), and 338b (*pdb* code 6L9Z [H1x] [ref. 22]) crystal structures (11 LH molecules in total; colored by variant type).

a

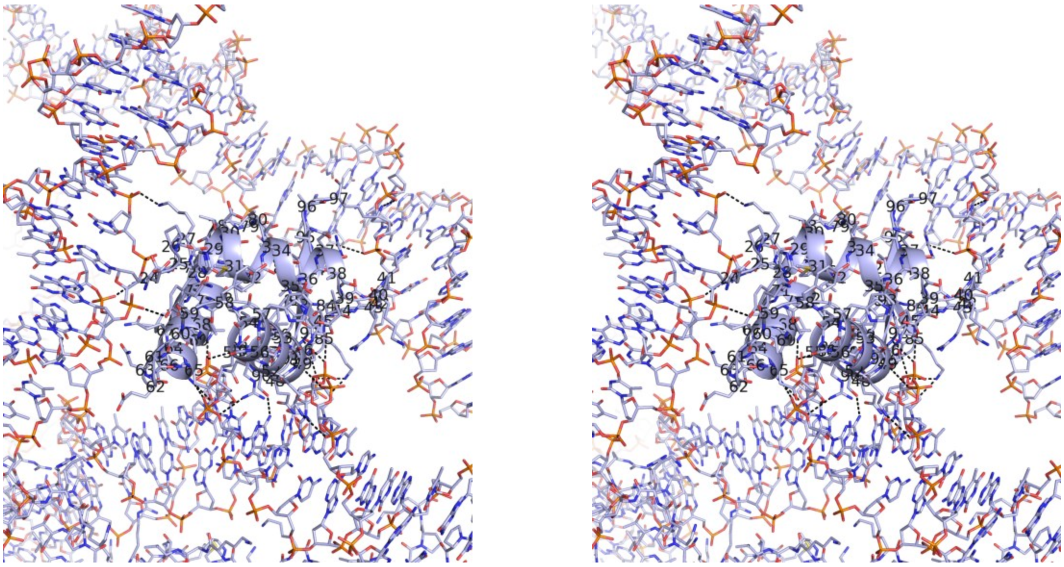

b

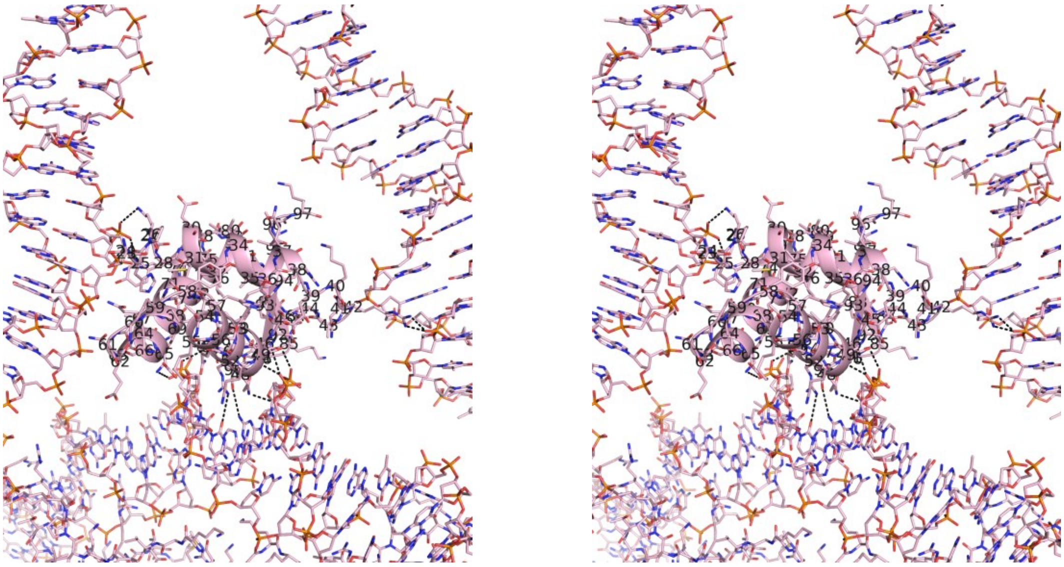

c

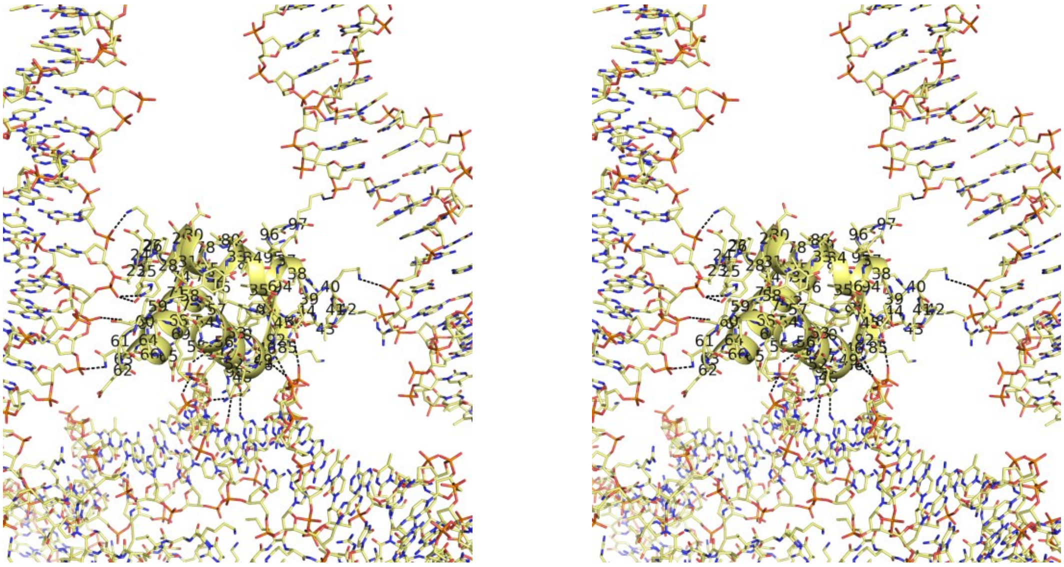

d

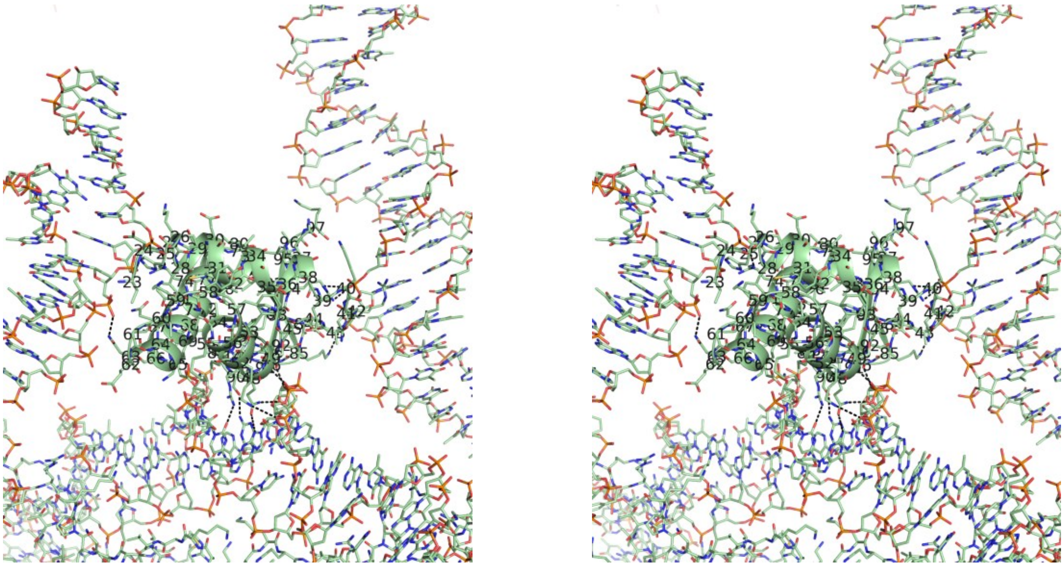

e

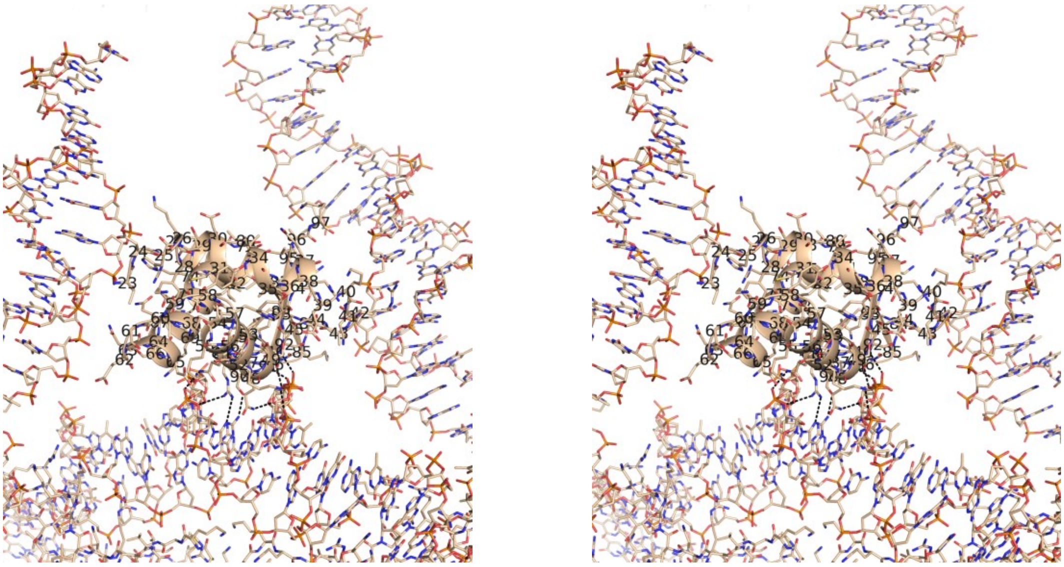

Supplementary Figure 6. Differential contact strength and stringency of linker histone-DNA interactions between the three globular domain binding interfaces, in stereo view. (**a**–**e**) Five unique on-dyad models from three different H1.0-dinucleosome assembly crystal structures (*pdb* codes 6LA9 [H1.0], 6LA2 $[H1.0]_2$, and 7COW $[H1.0]_2$, corresponding to the 349c, 343c, and 353e constructs, respectively; refs. 21 and 22) are rendered with distinct colors, as shown in Fig. 2 (see figure and legend for further information). LH residue numbers are displayed, and dashed lines indicate hydrogen bonding. (**a**) H1.0-349c structure. (**b**) H1.0-353e structure (H1.0, chain S). (**c**) H1.0-353e structure (H1.0, chain T). (**d**) H1.0-343c structure (H1.0, chain S). (**e**) H1.0-343c structure (H1.0, chain T). The α1/ℓ2/α3:linker DNA arm 1, ℓ1/s1/s2:linker DNA arm 2, and α2/α3/ℓ3:dyad interfaces are, respectively, at the left, right, and lower middle of the structures.

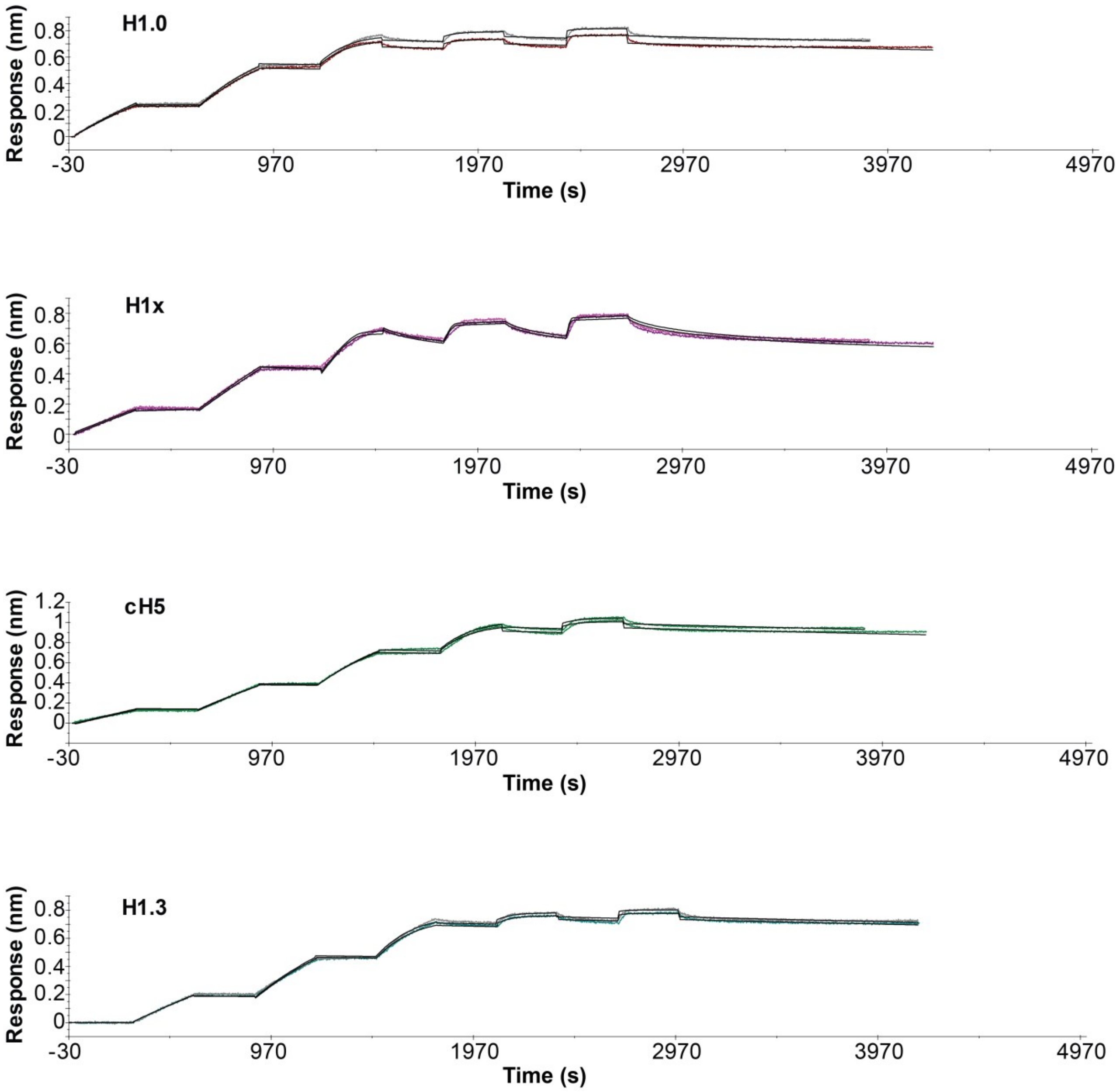

Supplementary Figure 7. Kinetic measurements of linker histone association with the 169a-nucleosome, based on bio-layer interferometry. Plots display real-time kinetic binding sensorgrams (coloured) along with fitted results (black trace; n = 2 biologically independent replicates for each of the four LH variants). Source data are provided as a Source Data file.

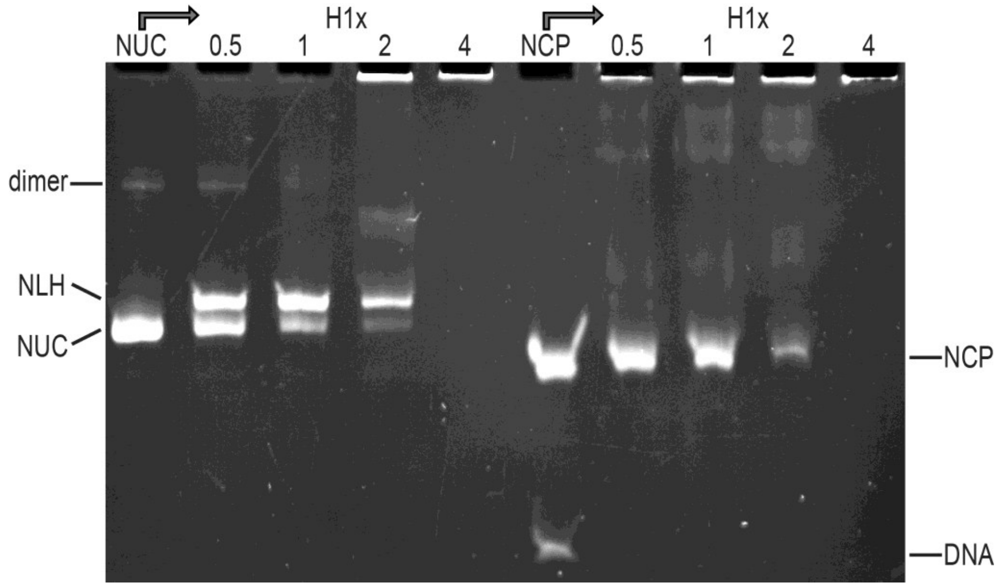

Supplementary Figure 8. Native polyacrylamide gel electrophoretic analysis of H1x association with 169a-nucleosome versus nucleosome core particle. Samples include nucleosome and NCP alone (NUC and NCP, respectively) or with the addition of 0.5, 1, 2, or 4 LH:nucleosome molar stoichiometry of H1x (NLH, nucleosome-LH complex). Source data are provided as a Source Data file.

a

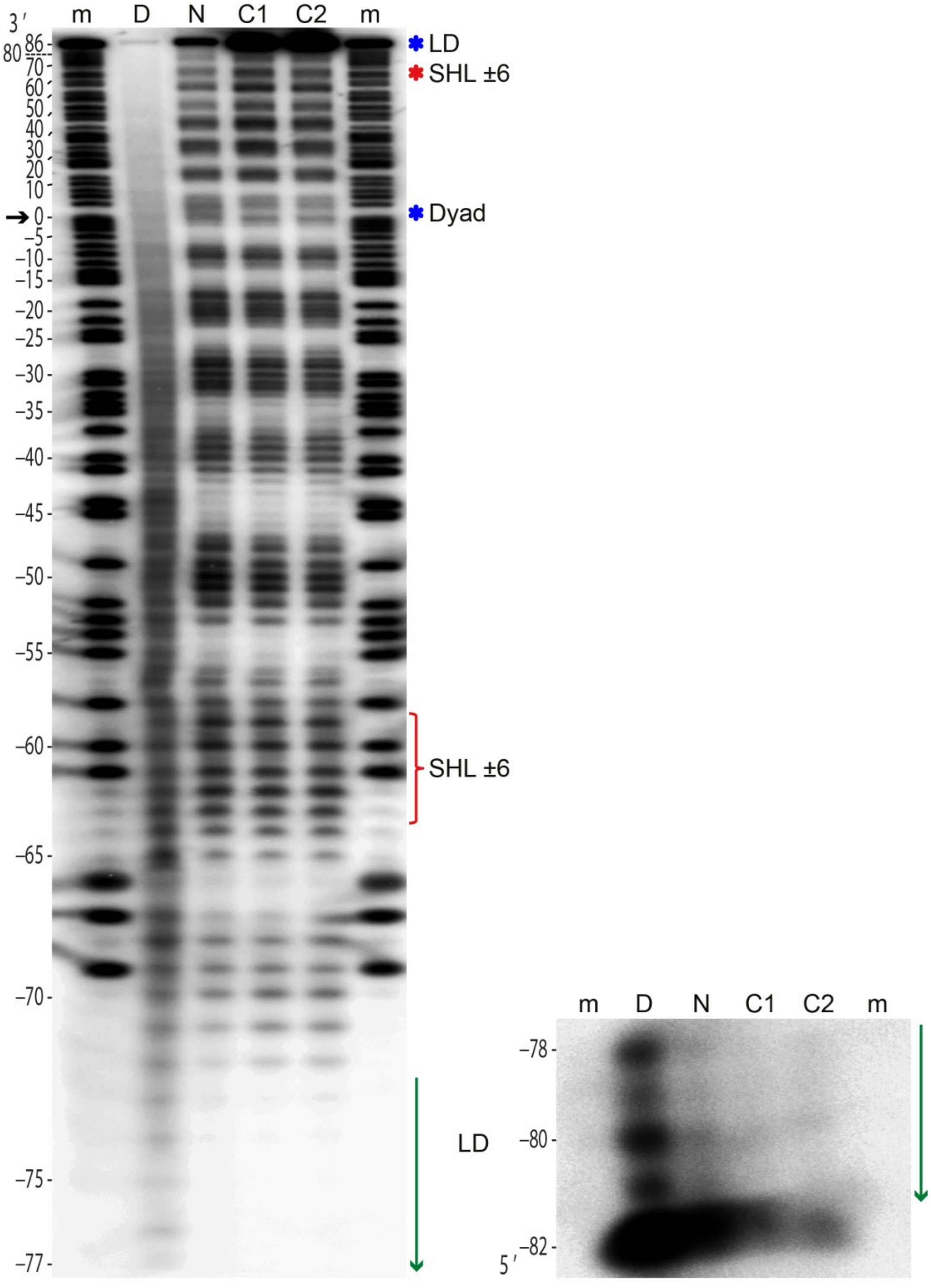

3′
m
D
N
C1
C2
m
86
80
70
60
50
40
30
20
10
0
−5
−10
−15
−20
−25
−30
−35
−40
−45
−50
−55
−60
−65
−70
−75
−77
LD
SHL ±6
Dyad
SHL ±6
m
D
N
C1
C2
m
−78
−80
−82
5′
LD

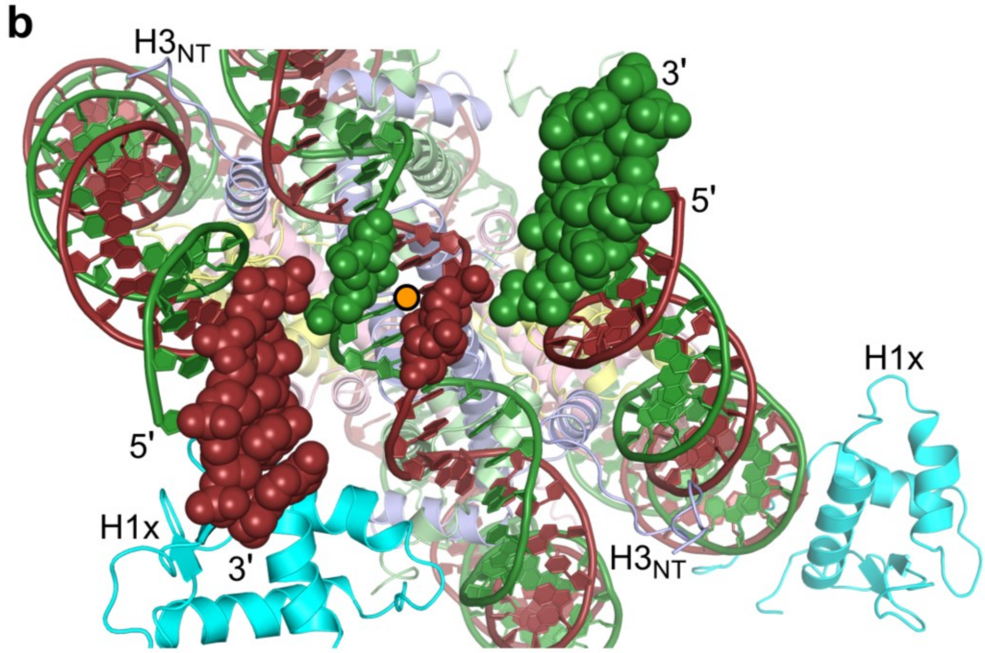

Supplementary Figure 9. H1x binding to the 169a-nucleosome in the solution state. (**a**) Hydroxyl radical footprinting analysis of DNA (D), nucleosome (N) or nucleosome-LH assembly with 1.0 (C1) or 1.2 (C2) LH:nucleosome molar stoichiometry of H1x. Purine sequencing markers are shown at the sides (m), with nucleotide position to the left of the gel. H1x-mediated protection from strand cleavage (blue asterisks) is apparent at the two pyrimidine nucleotides 3' to the central bp at the dyad and the 3' terminal region of the linker DNA (LD; apparent from the highly disproportionate quantity of remaining full-length DNA fragment). Conversely, H1x-mediated protection is not observed around the SHL ±6 regions (red asterisk and bracket), which would otherwise coincide with the main H1x-DNA interface seen in the H1x-169a and H1x-338b crystal structures. The 5' region of the linker DNA (green arrows) appears to be significantly protected in both the nucleosome and nucleosome-LH samples, which could be attributed to interactions with the $H3_{NT}$ and/or the $H2A_{CT}$. (**b**) H1x-169a crystal structure annotated to show the distinction with respect to H1x binding position in the solution state (apparently on-dyad). DNA sites protected by H1x in solution are shown in space filling and the view is looking directly down the dyad axis through the central bp (black circle). Source data are provided as a Source Data file.

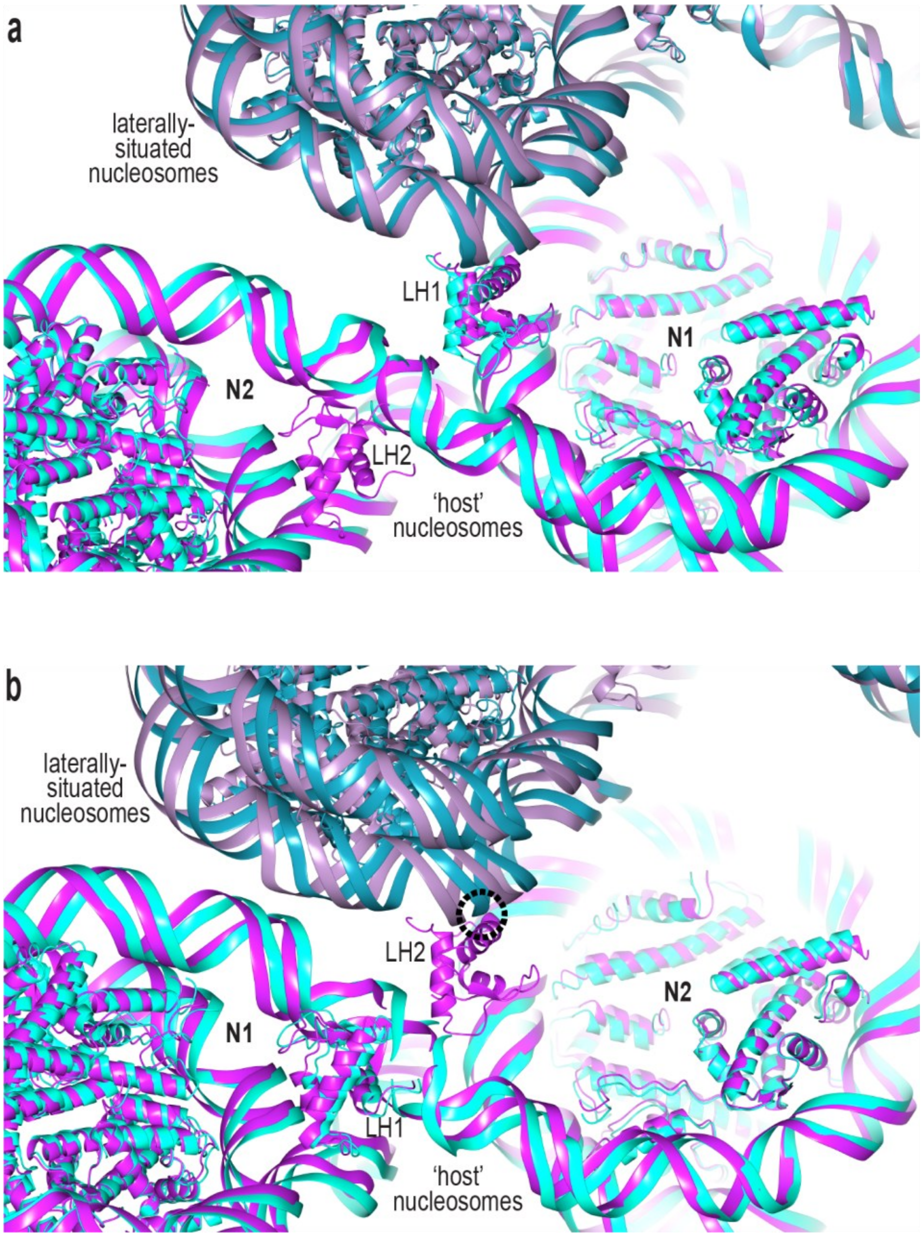

Supplementary Figure 10. Comparison of H1x binding in the 169a mononucleosome and 338b dinucleosome structures. (**a**,**b**) The 169 nucleosome dimer (magenta; asymmetric unit) is superimposed onto the 338 dinucleosome (cyan; asymmetric unit). The laterally situated nucleosomes are shown in dark magenta (169) and dark cyan (338). Whereas H1x is bound at both the LH1 and LH2 locations in the 169 structure, it occupies only the LH1 location in the 338 structure. This is a consequence of favourable interactions with both the host and lateral nucleosomes for the LH1 location (a), but unfavourable positioning of the lateral-relative-to-host nucleosomes for the LH2 location (b; notably clash relative to minor groove insertion of $\alpha3$; area indicated with circle).

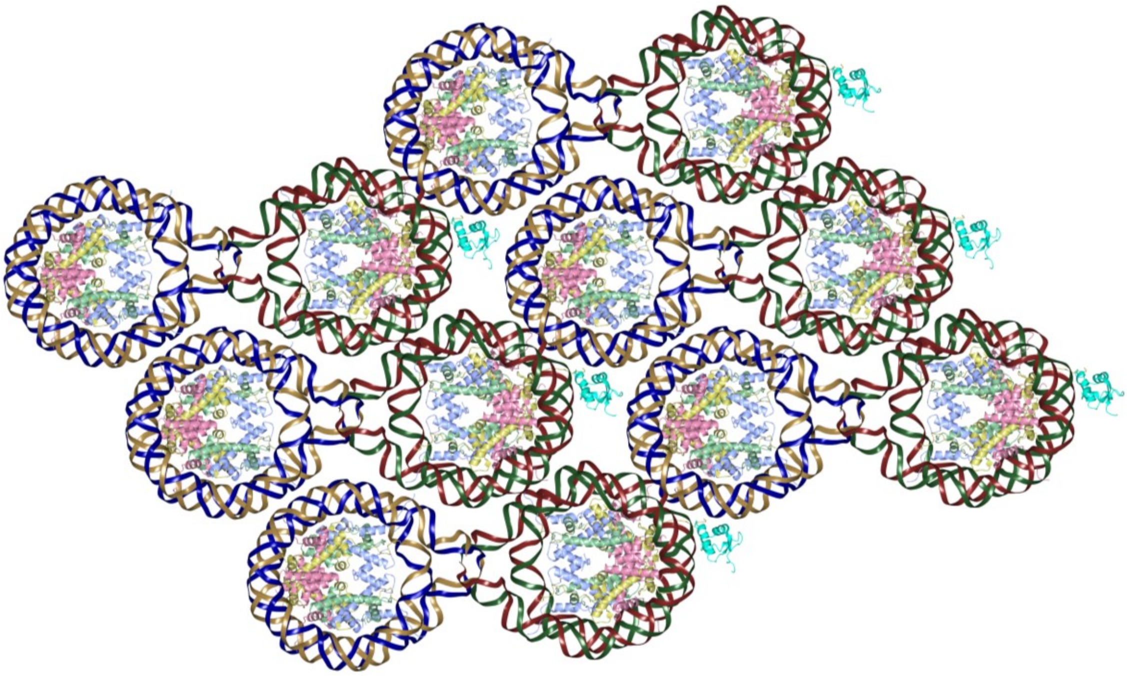

Supplementary Figure 11. Internucleosomal contacts in the H1.0-169a crystal polymorph corresponding to the L1b mode. The view is of a single nucleosome layer, along the 2-fold screw (2_1) axes of the crystal (color scheme as in Fig. 1; H1.0, cyan). The lattice differs somewhat relative to the alternate H1.0-169a crystal packing configuration associated with the L1 mode, which is nearly identical to that seen for the H1x-169a crystals (also L1 mode; see Fig. 1c).

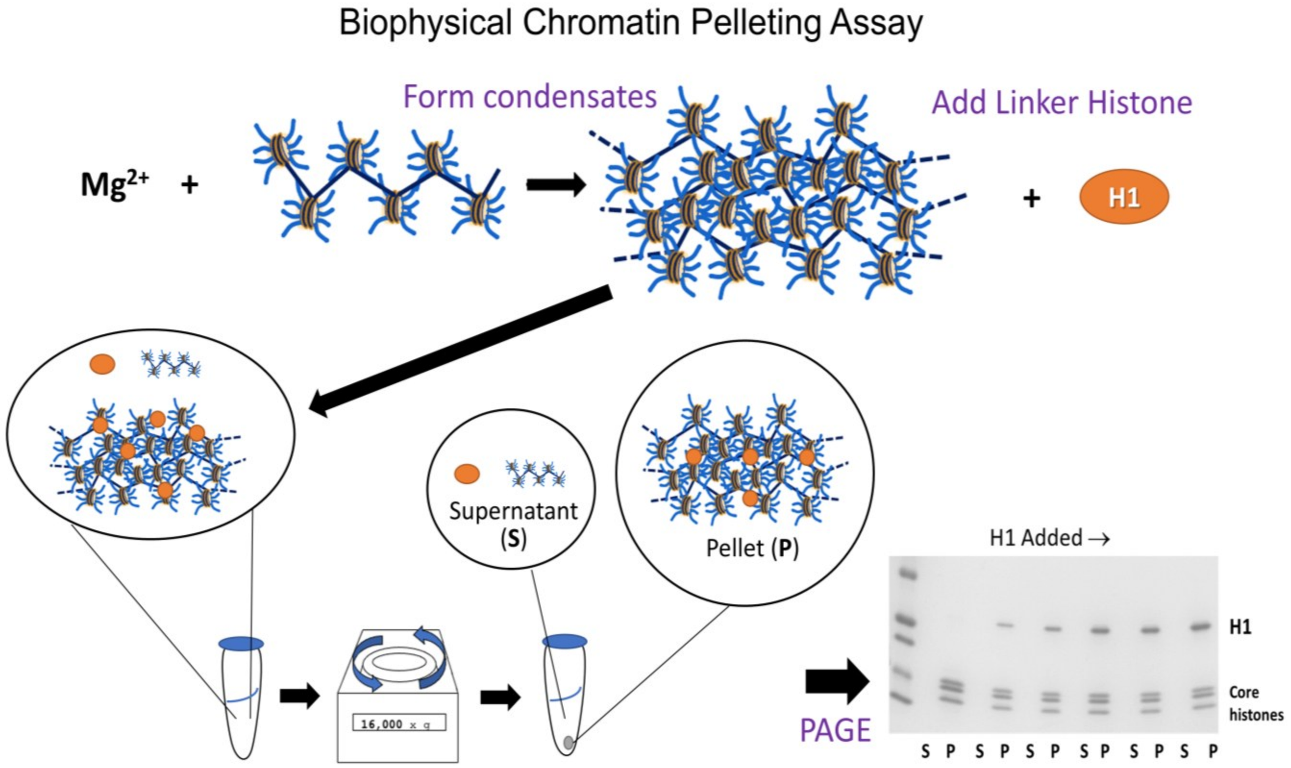

Supplementary Figure 12. Scheme for the Pelleting Assay used to quantify H1 binding to the nucleosome array condensates. Subsequent to SDS-PAGE analysis, the amounts of H1 and nucleosome are quantified by band densitometry and H1:nucleosome stoichiometry is established by comparison with loaded standards (see Supplementary Figure 13).

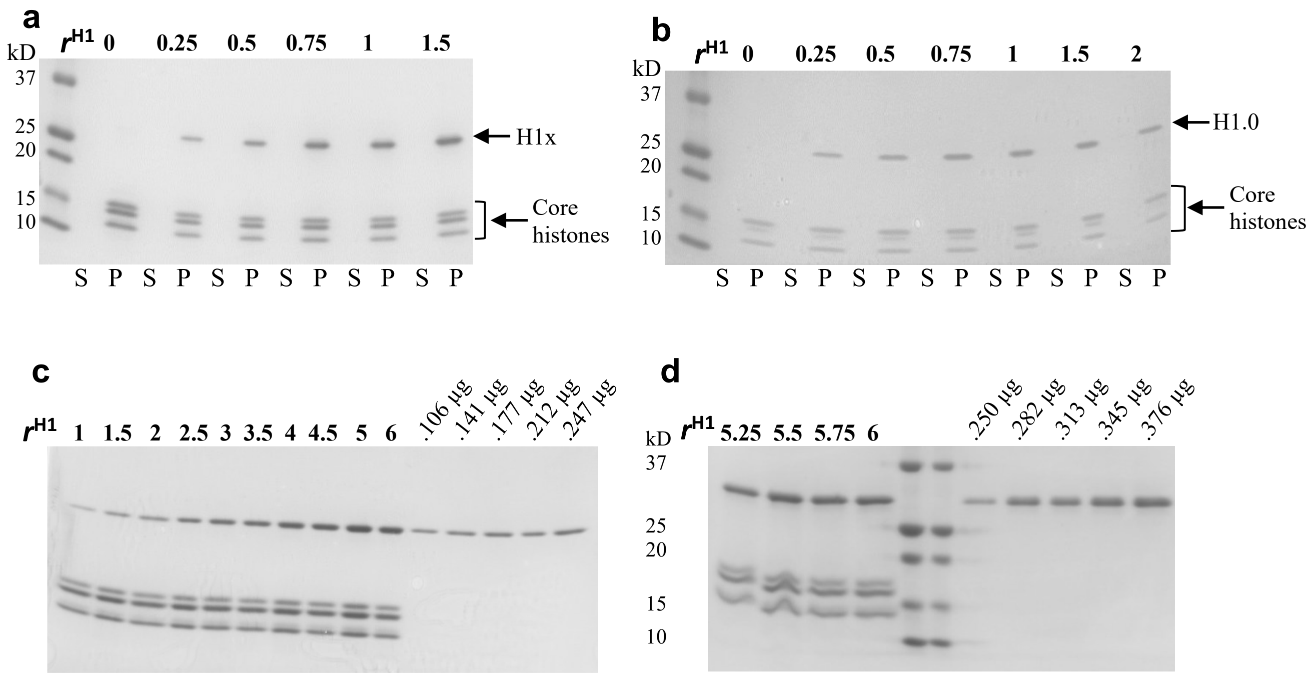

Supplementary Figure 13. Application of pelleting assay to quantify linker histone:nucleosome binding stoichiometry in nucleosome array condensates. **(a,b)** SDS-PAGE gels showing results of pelleting assays for nucleosome array incubated with H1x (a) or H1.0 (b). Precision Plus All Blue protein standard (Goldbio, St Louis, MO, USA) with band sizes in kD are shown. S denotes supernatant fractions, P corresponds to pellet fractions, and bands for H1 and core histones are indicated. **(c,d)** Representative SDS-PAGE gels showing the pellet fractions correspond to pelleting assays for nucleosome array incubated with H1 (left side of gels) alongside standards (right side) for quantitation. The H1:nucleosome molar input ratios, r^{H1}, are indicated above each corresponding lane on the left side of the gels. The amount, in μg, of H1 standard loaded is shown above each corresponding lane on the right side of the gels (protein standard, d, middle). Source data are provided as a Source Data file.

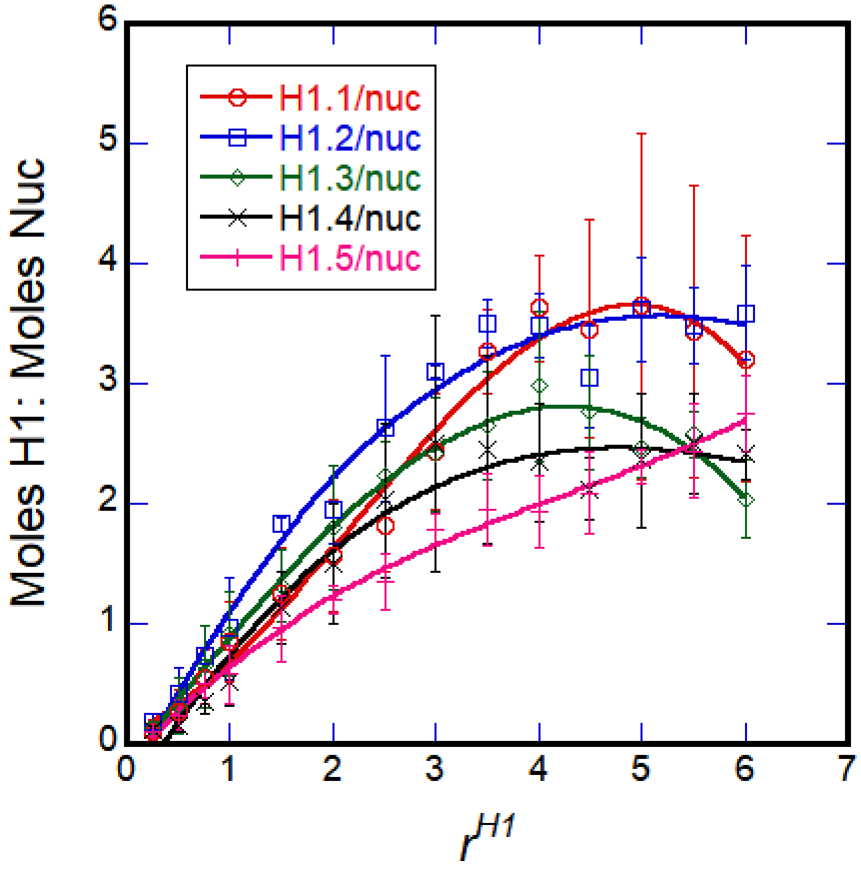

Supplementary Figure 14. Linker histones bind multivalently to nucleosome array condensates. Condensate binding curves for the DNA replication-dependent linker histone variants, H1.1–H1.5. The x-axis coincides with the H1:nucleosome molar stoichiometry of H1 incubated with the array (r^{H1}), and the y-axis corresponds to the H1:nucleosome molar stoichiometry quantified for the amount of LH bound in the array condensates (error bars represent standard deviation, n ≥ 3 biologically independent replicates). Source data are provided as a Source Data file.

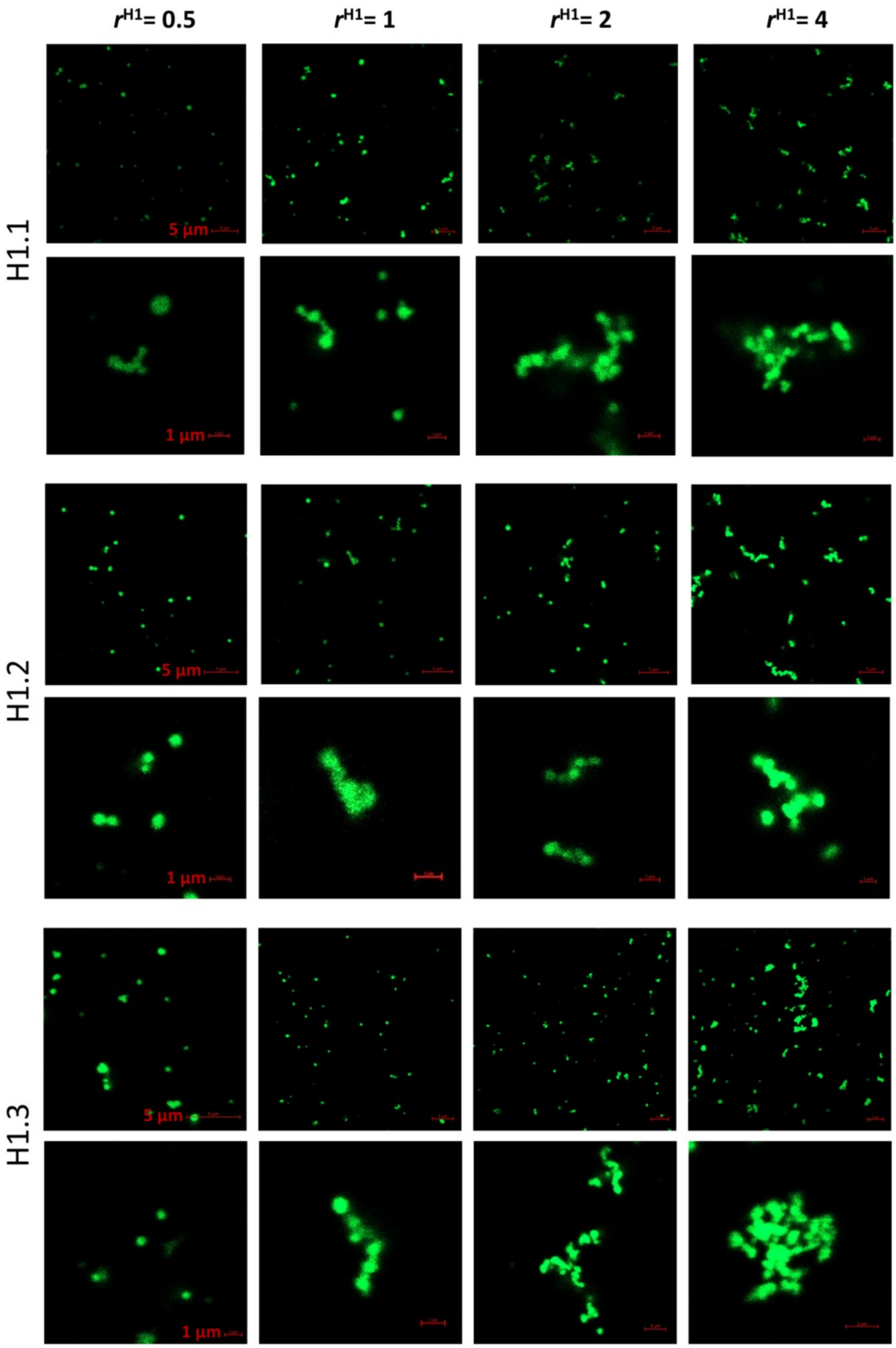

Supplementary Figure 15. Continued on the following page

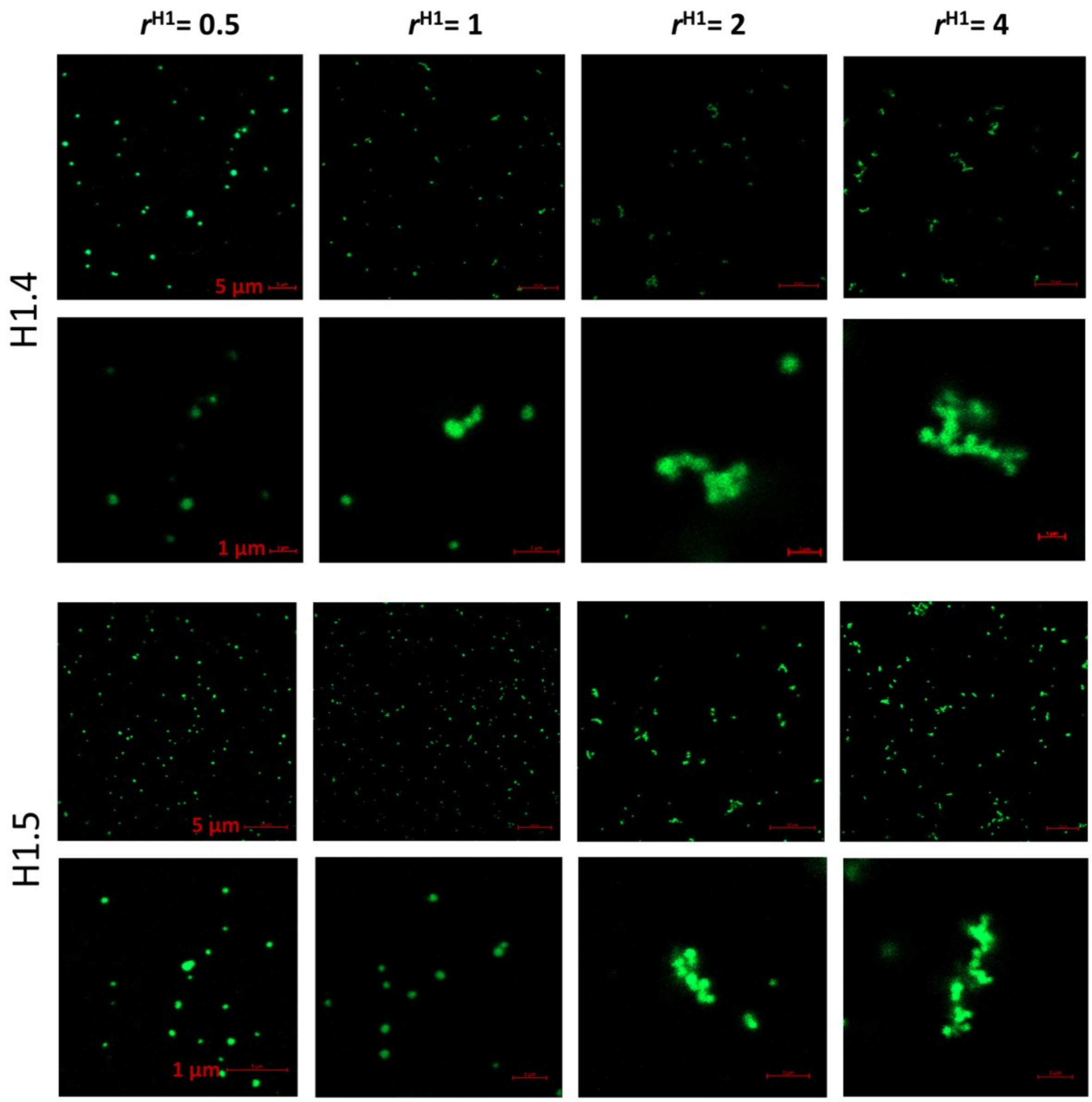

Supplementary Figure 15. Linker histones stabilize complex fiber-fiber interactions. Fluorescence microscopy imaging of array condensates incubated with varying H1:nucleosome molar stoichiometry (r^{H1}) of either H1.1, H1.2, H1.3, H1.4, or H1.5 (n = 3 biologically independent replicates). Source data are provided as a Source Data file.

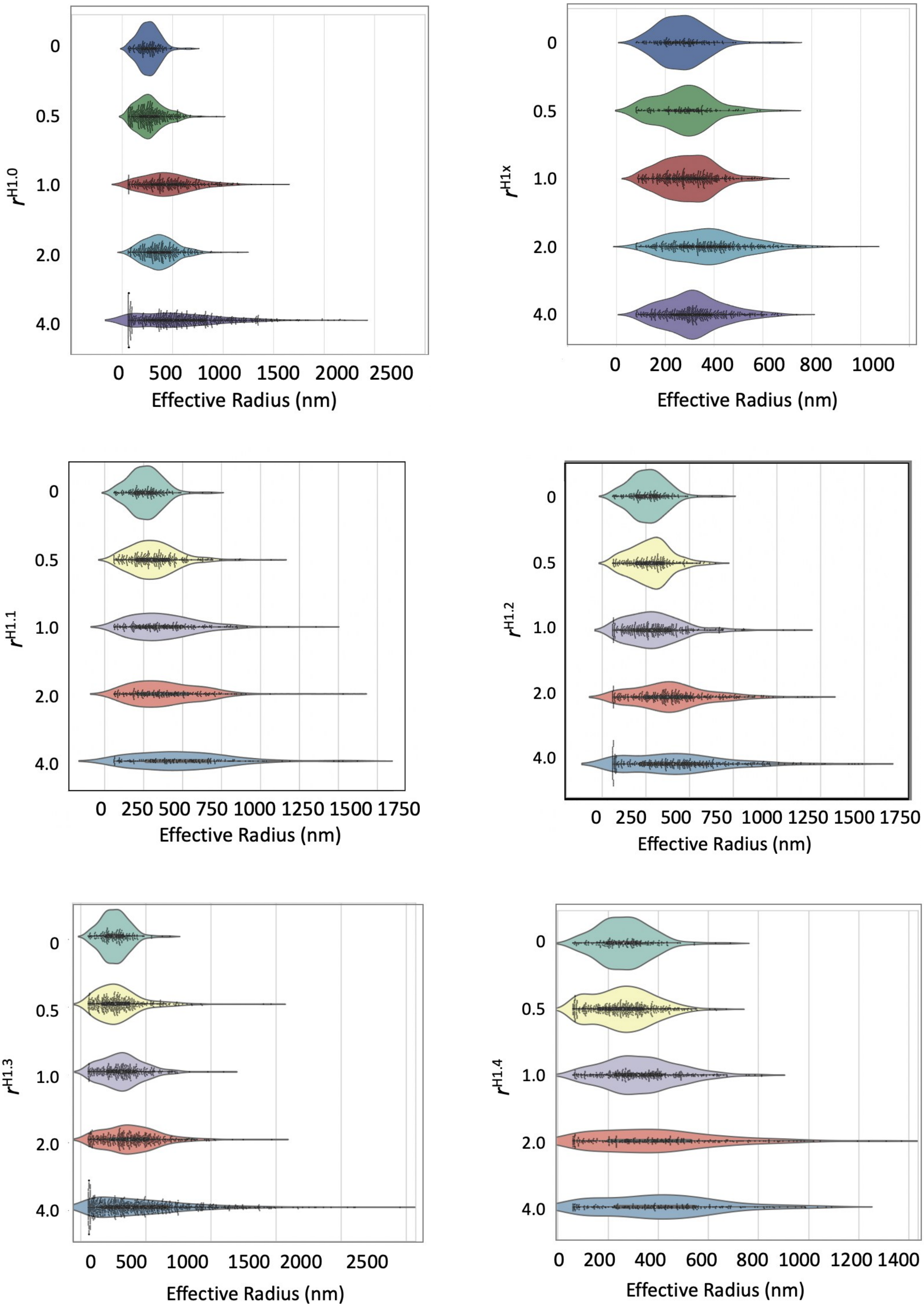

0
0.5
1.0
2.0
4.0
$r^{H1.0}$
0 500 1000 1500 2000 2500
Effective Radius (nm)
r^{H1x}
0 200 400 600 800 1000
Effective Radius (nm)
$r^{H1.1}$
0 250 500 750 1000 1250 1500 1750
Effective Radius (nm)
$r^{H1.2}$
0 250 500 750 1000 1250 1500 1750
Effective Radius (nm)
$r^{H1.3}$
0 500 1000 1500 2000 2500
Effective Radius (nm)
$r^{H1.4}$
0 200 400 600 800 1000 1200 1400
Effective Radius (nm)

Supplementary Figure 16. Continued on the following page

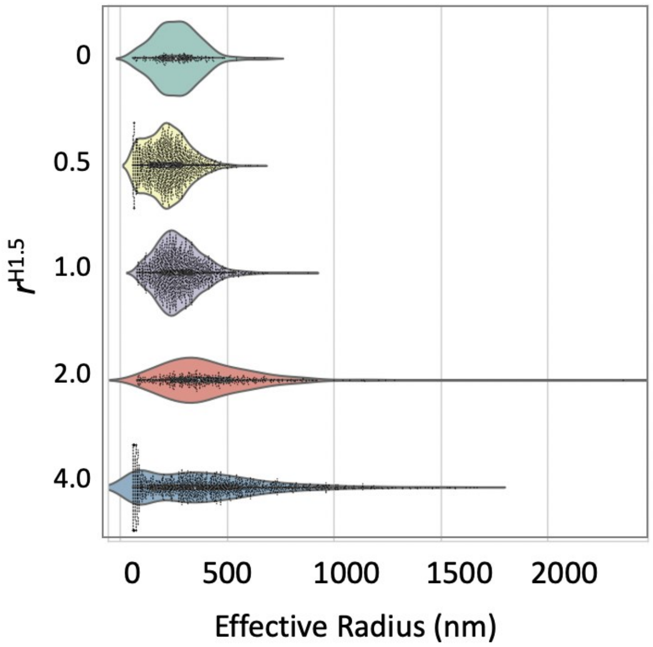

Supplementary Figure 16. Linker histones stabilize complex fiber-fiber interactions in nucleosome array condensates. Violin plots calculated from fluorescence microscopy imaging of nucleosome array condensates show the distribution of condensate ratio as a function of H1:nucleosome molar stoichiometry (r^{H1}; across the seven somatic H1 variants). Source data are provided as a Source Data file.

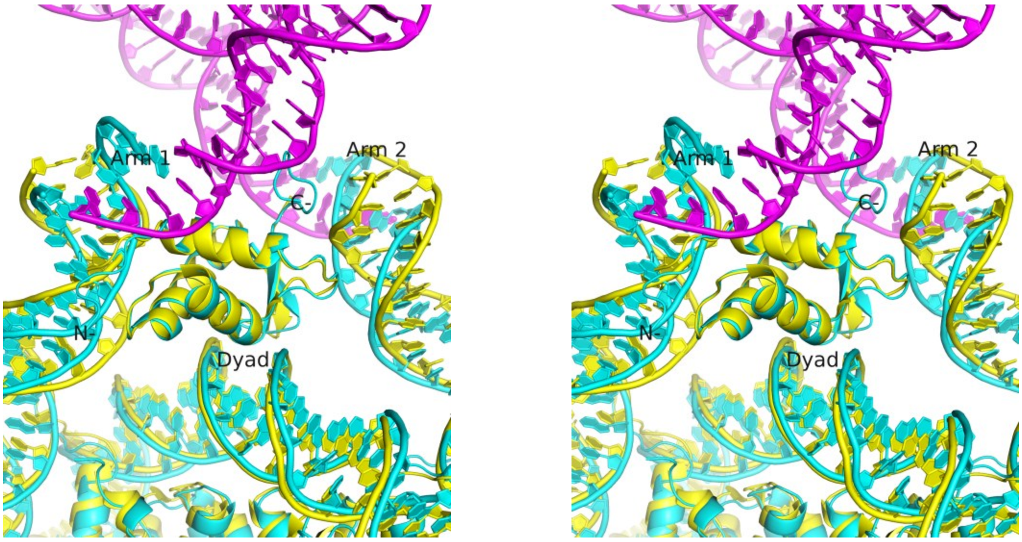

Supplementary Figure 17. Comparison of the on-dyad H5 association in the H5-169a structure (cyan/magenta) with that of the H5 globular domain bound to a 167 bp nucleosome (yellow), in stereo view. For the H5-169a nucleosome dimer, the H5 (N-, N-terminal residue in model; C-, C-terminal residue in model) and the nucleosome hosting this on-dyad bound LH are coloured cyan, whereas the other (paired) nucleosome is coloured magenta. The $H5_{GD}$-167 bp-nucleosome model (*pdb* code 4QLC) is from Zhou *et al.*, 2015 (ref. 28) and is coloured yellow. The two structures were superimposed by least-squares fitting of the respective H5 globular domains, thereby emphasizing the pronounced distinctions in the structure/positioning of the linker DNA arms (Arm 1, interaction with H5-α1/ℓ2/α3 interface; Arm 2, interaction with H5-**ℓ**1/s1/s2 interface) relative to the nucleosome centre (Dyad, interaction with H5-α2/α3/ℓ3 interface).
