# Supplementary material for: Linker histones consolidate heterogenous nucleosome fiber contacts by linking together multiple nucleosomes
Source: Nat Commun. 2026 Mar 5;17:3807. doi: 10.1038/s41467-026-69842-x (PMC13111611; doi:10.1038/s41467-026-69842-x)
Supplement: Supplementary file 2 — Description of Additional Supplementary Files [file 41467_2026_69842_MOESM2_ESM.pdf]

Description of Additional Supplementary Files

Supplementary Data 1. List of linker histone-DNA hydrogen bonding interactions for the nucleosome assemblies with H1x, H1.0, H1.3, and H5.
